# A Narrative Review of the Effects of Citrus Peels and Extracts on Human Brain Health and Metabolism

**DOI:** 10.3390/nu14091847

**Published:** 2022-04-28

**Authors:** Kentaro Matsuzaki, Akira Nakajima, Yuanqiang Guo, Yasushi Ohizumi

**Affiliations:** 1 Department of Environmental Physiology, Faculty of Medicine, Shimane University, En-ya-cho 89-1, Izumo 693-8501, Japan; 2 Department of Applied Biology and Food Sciences, Faculty of Agriculture and Life Science, Hirosaki University, 3 Bunkyo-cho, Hirosaki 036-8561, Japan; anakajim@hirosaki-u.ac.jp; 3 State Key Laboratory of Medicinal Chemical Biology, College of Pharmacy, Tianjin Key Laboratory of Molecular Drug Research, Nankai University, Tianjin 300350, China; victgyq@163.com; 4 Kansei Fukushi Research Institute, Tohoku Fukushi University, 6-149-1 Kunimigaoka, Aoba, Sendai 989-3201, Japan; oyyaa2@crux.ocn.ne.jp

**Keywords:** citrus peel extracts, brain health, Alzheimer’s disease, nobiletin, polymethoxylated flavone, flavanone, clinical trials, obesity, cardiovascular function

## Abstract

As life expectancy increases, age-associated diseases such as Alzheimer’s disease (AD) become a major health problem. The onset of AD involves neurological dysfunction due to amyloid-β accumulation, tau hyperphosphorylation, oxidative stress, and neuroinflammation in the brain. In addition, lifestyle-related diseases—such as dyslipidemia, diabetes, obesity, and vascular dysfunction—increase the risk of developing dementia. The world population ages, prompting the development of new strategies to maintain brain health and prevent the onset of dementia in older and preclinical patients. Citrus fruits are abundant polymethoxylated flavone and flavanone sources. Preclinical studies reported that these compounds have neuroprotective effects in models of dementia such as AD. Interestingly, clinical and epidemiological studies appear to support preclinical evidence and show improved cognitive function and reduced associated disease risk in healthy individuals and/or patients. This review summarizes the recent evidence of the beneficial effects of citrus peels and extracts on human cognition and related functions.

## 1. Introduction

As the world population ages, the number of patients with cognitive disorders such as Alzheimer’s disease (AD) and cardiovascular dementia increases [1,2]. AD is one of the most common types of dementia and a progressive neurological illness, mainly in older people [1]. The research from the last 30 years leaves no doubt regarding the role of amyloid-beta (Aβ) neurotoxicity and tau hyperphosphorylation in AD development. In line with these findings, oxidative stress, neuroinflammation, cerebral vascular dysfunction, cholinergic neurodegeneration, and cerebral amyloid angiopathy are also associated with AD development [3,4,5,6,7,8,9,10]. Besides, lifestyle-related diseases—such as diabetes, dyslipidemia, and obesity—are associated with increased AD risk [11,12,13,14]. In recent decades, clinical AD treatments have included the acetylcholinesterase inhibitors donepezil, rivastigmine, and galantamine, and the non-competitive *N*-methyl-d-aspartate receptor antagonist memantine [15]. These drugs temporarily reduce cognitive dysfunction in AD patients and have some effect on AD [16]. Most recently, the Food and Drug Administration of the United States of America approved aducanumab, a particularly promising drug acting directly on Aβ pathology [17,18]. Besides these therapeutic agents, new neurodegenerative disease prevention strategies are in development, including nutritional interventions, adjuvants, and complementary and alternative medicine approaches [19,20,21,22,23].

**Figure 1:**
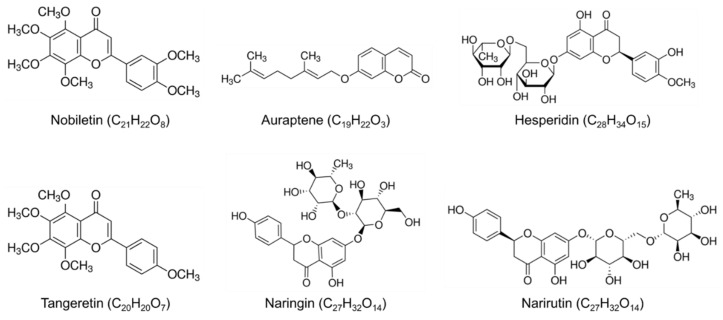
Chemical structure of compounds abundant in citrus peels, extracts, and juices.

Several natural resources contain bioactive substances with potential applications in the treatment and prevention of neurodegenerative and/or lifestyle-related diseases [24,25,26]. Citrus fruits contain large amounts of polymethoxylated flavones (PMFs), such as nobiletin and tangeretin; and flavanones, such as naringin, hesperidin, and narirutin [27] (Figure 1). The content of these flavonoids varies among citrus varieties. For example, nobiletin is present at high concentrations in species of the *Acrumen* and *Aurantium*, but not in the *Fortunella* and *Poncirus* species. In general, citrus flavonoids are more abundant in the pericarp than in the edible parts, e.g., nobiletin and hesperidin are most abundant in the albedo (endocarp) and flavedo parts [27]. Although citrus flavonoid content varies by variety and lot, the flavonoid content in the peels of ponkan (*C. reticulata*), is as follows; nobiletin 110 mg, tangeretin 124 mg, hesperidin 1370 mg, and narirutin 44.2 mg per 100 g fresh weight, respectively [27].

Numerous preclinical studies in animals and/or cultured cells demonstrated the beneficial effects of citrus flavonoids in various neurodegenerative disease [28,29] and diabetes/obesity models [30,31,32]. Indeed, citrus flavonoids exert neuroprotection and prevent dementia-related cognitive decline by acting on their pathological features and related mechanisms, including Aβ/tau pathology, inflammation, oxidative stress, apoptosis, neurodegeneration, dyslipidemia, and cardiac and synaptic dysfunction. Citrus flavonoids also exert neuroprotective effects in animal models of various neurological disorders through oral, subcutaneous, and intraperitoneal administration [28,29]. Additionally, these compounds and their metabolites readily cross the blood–brain barrier [33,34,35]. These basic studies prompted several clinical trials on intake of citrus components and human neurological function, lipid metabolism, and circulatory function. Interestingly, the clinical and epidemiological studies showed improved cognitive function and reduced disease risk in patients and healthy subjects, in line with the preclinical studies. This paper reviews the current literature on clinical and epidemiological investigations on the beneficial effects of citrus components on brain health and related functions. 

## 2. Citrus Compounds on Brain Health

Citrus PMFs, such as nobiletin and tangeretin, exerted beneficial effects on cognitive function in numerous experimental models—e.g., AD, Parkinson’s disease, and cardiovascular dementia [36,37,38,39,40]—by modulating pathological features such as Aβ/tau pathology, oxidative stress, and neuroinflammation and improving synaptic plasticity in several experimental models [41,42,43,44]. Besides, flavanones such as hesperidin, naringin, and narirutin exerted neuroprotection in several neurodegenerative disorder models [45,46,47]. Human intervention studies based on these results are currently taking place.

In general, animal and in vitro studies commonly use pure flavonoids, while clinical trials and epidemiological surveys use citrus peel powder, tablets, capsules, extracts, or juices. Accumulating evidence suggests that acute and/or chronic consumption of citrus compounds benefits brain health (Table 1).

### 2.1. Nobiletin-Rich Citrus Peel Extract Improves Cognitive Function

A double-blind, randomized controlled study assessed the effect of Nobilex^®^—containing *C. depressa* peel dried powder extract (equivalent to 10.0 mg nobiletin and 5.8 mg tangeretin), *P. japonicum* dried leaf powder (33.3 mg), and *K. parviflora* dried root powder (126.7 mg)—on cognitive function [48]. They compared healthy older Japanese individuals (*n* = 108) receiving Nobilex^®^ once a day for 16 weeks (mean age 73.3) to a placebo group (mean age 72.2). The authors assessed cognitive function using the Japanese version of the Wechsler Memory Scaled-Revised (WMS-R). The treated group had significantly higher “general memory” and “visual memory” scores on the WMS-R scale than the placebo group. Besides, the treated group had a significantly greater difference in total WMS-R scores than the placebo group. Additionally, in an age-stratified analysis of the WMS-R test, the authors observed similar changes in participants aged 74 years or less and the overall population. Among participants with Mini-Mental State Examination (MMSE)-J scores of 24 to 28, the “figural memory” Nobilex^®^-treated subjects had a significantly greater WMS-R subscale score than the placebo group. No serious adverse effect was observed. These results suggest that test foods containing nobiletin ameliorate memory dysfunction in healthy older adults [48].

A recent study documented the synergistic effects of citrus peel powder and *perilla* seed oil (PO) on cognition [49]. PO is rich in α-linolenic acid (ALA) and improves cognitive function and mental health in healthy and older adults [61,62]. Healthy older individuals (aged 60–85) received supplements containing either PO alone or PO with nobiletin-rich immature ponkan powder (PP) for 12 months. The PO group received capsules containing 1.47 mL (0.88 g of ALA) of PO daily, and the POPP group received capsules containing both 1.47 mL of PO and 1.12 g of PP (containing 2.91 mg of nobiletin). The authors evaluated cognitive function through MMSE, Hasegawa Dementia Scale-Revised (HDS-R), and the Japanese version of the Montreal Cognitive Assessment (MoCA-J). The 12-month intervention significantly increased the MMSE score in the POPP group. Besides, the POPP group had significantly higher scores in indicators such as MMSE sub-items “Attention and Calculation” and “Language”, HDS-R sub-item “Serial Subtraction”, and Moca-J sub-item “Short-Term Memory”. Moreover, POPP markedly increased serum brain-derived neurotrophic factor (BDNF) levels and biochemical antioxidant capacity. Finally, POPP did not affect the subjects’ blood pressure or blood biochemical levels and did not cause allergies or other physical reactions. These results suggest that long-term POPP intake enhances BDNF and, potentially, antioxidant levels as well as preventing age-related cognitive decline in healthy older individuals [49].

A clinical trial evaluated the anti-dementia effect of nobiletin-rich *Citrus reticulata* peel extract on AD patients taking donepezil [50]. The patients had been taking donepezil (5 mg) for more than a year and had an MMSE score of 25 or less (mild to moderate cognitive impairment). The patients (*n* = 11) were randomly divided into a control group (*n* = 5) and an intervention group (*n* = 6). The intervention group ingested extract (obtained from 30 g of citrus peels boiled in 500 mL of water and concentrated to 300 mL) three times daily for one year. Cognitive function was evaluated using the MMSE and the Japanese version of the AD Assessment Scale-Cognitive Subscale (ADAS-J cog). Comparing the cognitive scores before and after the intervention revealed that the MMSE score had decreased, and the ADAS-J cog score had increased in the control group. Meanwhile, the intervention group had unchanged scores. Thus, long-term intake of citrus peel extract suppressed cognitive decline in AD patients. Moreover, long-term citrus peel ingestion caused no apparent side effects. These results suggest that long-term intake of nobiletin-rich citrus peel extract prevents AD progression [50]. 

Finally, nobiletin-rich citrus peel extracts may improve cognitive function in the elderly and AD patients by antioxidant and anti-inflammatory effects, activating signaling pathways related to memory formation (i.e., the cAMP/PKA/CREB/BDNF pathway), and improving synaptic plasticity in the cortex and hippocampus [28,29,63].

### 2.2. Effect of Flavanone-Rich Citrus Juices on Cognitive Function

Preclinical studies indicated that flavanones, such as hesperidin and narirutin, improve cognitive impairments induced by oxidative stress, inflammation, and ischemia [45,46,47]. These flavanones have also been reported to improve cognitive function through various mechanisms, including increasing BDNF levels and improving neurological function [45,46,47].

The benefits of flavanone-rich citrus juices for cognitive function in humans have been tested. Kean et al. (2015) investigated the long-term (8-week) consumption of flavanone-rich orange juice on cognitive function in 37 healthy individuals (aged 60–81 years) [51]. The study was a randomized, double-blind clinical trial with a crossover design. Participants consumed 500 mL of flavanone-rich orange juice daily for eight weeks (305–549 mg hesperidin and 60 mg narirutin/day). Compared with a placebo, chronic flavanone-rich orange juice intake improved the global cognitive score. Moreover, long-term flavanone-rich beverage consumption improved “recall” significantly as well as executive function, albeit marginally (*p* = 0.06). Neither the mood of the participants nor their blood pressure changed [51].

The acute effect of flavanone-rich orange juice on neurological responses has also been assessed [52,53]. In 24 healthy middle-aged adults (30–65 years), a flavonoid-rich beverage (272–220.46 mg hesperidin, 34.54 mg narirutin, and 17.14 mg other flavonoids) improved the cognitive scores at 2 and 6 h post-consumption [52]. Besides, consuming flavonoid-rich orange juice increased performance in a simple finger tapping test after 2 h and continuous performance task test after 6 h. It also increased subjective arousal levels and marginally improved global cognitive ability. Interestingly, the significant improvement observed in cognition and subjective arousal levels 6 h after ingestion are consistent with flavanone metabolites peak 5–7 h after ingestion [64]. Lamport et al. (2016) also investigated the acute effect of a flavanone-rich beverage on neurological functions [53]. This single-blind, randomized, crossover trial used a commercially available flavanone-rich beverage (equivalent 42.15–70.5 mg hesperidin, 17.25 mg naringin, 6.75 mg narirutin, and 4.3 mg caffeic acid). This study measured cerebral blood flow (CBF) using functional magnetic resonance imaging to assess blood flow to the brain and specific brain areas responding to ingestion. Participants (aged 18–30 years) either underwent cognitive assessment 2 h after ingestion (*n* = 28) or CBF measurements 2 h and 5 h after ingestion (*n* = 16). Flavanone-rich juice significantly enhanced brain perfusion in the inferior frontal gyrus and right middle frontal gyrus of the right hemisphere 2 h after consumption. Interestingly, the improvement in the numeric symbol substitution score (a measure of executive function) after 2 h correlated with increased regional perfusion in the inferior frontal gyrus, an area involved in executive performance [65]. Thus, both chronic and acute flavanone-rich beverage intake improved neurological function.

Kawachi Bankan (*Citrus kawachiensis*) is a citrus fruit, and its extract contains not only PMFs (e.g., 3,5,6,7,8,3′,4′-heptamethoxyflavone) and flavanones (e.g., naringin) but also large amounts of auraptene (7-geranyloxycoumarin, AUR). Preclinical studies showed that these compounds improved cognitive dysfunction caused by inflammation and ischemia [66,67,68]. A double-blind, randomized controlled trial including 82 healthy older people assessed the effect of Kawachi Bankan extract on cognition [54]. Participants received either test juice (equivalent AUR 6 mg/day) or a placebo beverage (AUR 0.1 mg/day) for 24 weeks. The authors evaluated cognitive functions before and after intervention using the 10-word recall test of the mild cognitive impairment screening test (MCI screen). The treated group achieved better scores than the placebo group. Thus, the continuous intake of the AUR-rich Kawachi Bankan extract suppressed cognitive decline in older adults, suggesting that this test beverage improves cognitive function in older adults [54]. AUR has been reported to increase BDNF production and may be involved in cognitive improvement [68].

### 2.3. Citrus Consumption and Cognitive Function: Evidence from Cohort Studies

Several epidemiological studies have reported the benefits of chronic citrus components ingestion on cognitive function. Zhang et al. (2017) investigated the association between dementia incidence and daily citrus consumption in 13,373 subjects (aged 65 years or more) [55]. They used a Food Frequency Questionnaire (FFQ) combined with the Japanese Long-Term Care Insurance database over 5.7 years. The study revealed an inverse correlation between daily citrus fruit intake and dementia onset. The hazard ratio for dementia showed that people consuming citrus less than twice a week had a higher risk of developing dementia than those consuming citrus 3–4 times/week or almost every day [55]. Thus, a daily intake of citrus components may reduce the risk of developing dementia.

Yeh et al. (2021) also investigated the association between long-term dietary intake of flavonoids, such as citrus juice, and cognitive decline [69]. They gathered data regarding 49,493 women from the Nurses’ Health Study (NHS) conducted from 1984 to 2006, and 27,842 men from the Health Professionals Follow-Up Study (HPFS) conducted from 1986 to 2002. For the NHS, they measured long-term average dietary consumption from seven repeated semiquantitative FFQs (SFFQs) and evaluated cognitive decline in 2012 and 2014. For the HPFS, they speculated average dietary consumption from five repeated SFFQs and evaluated cognitive decline in 2008 and 2012. The authors revealed that a higher total flavonoids intake was correlated with lower odds of cognitive decline after adjustment for age, total energy intake, specific dietary factors, and major nondietary factors. Comparing the highest and the lowest quintiles of total flavonoid consumption revealed that the pooled multivariable-adjusted odds ratio of three-unit increments in cognitive decline was 0.81 (95% confidence interval 0.76, 0.89). The pooled results indicated clear associations between reduced cognitive decline and flavones (odds ratio 0.62 (95% CI 0.57, 0.68)), flavanones (odds ratio 0.64 (0.58, 0.68)), and anthocyanins (odds ratio 0.76 (0.72, 0.84)). Many flavonoid-rich foods—such as citrus juices, oranges, grapefruits, pears, apples, celery, bananas, and peppers—were clearly correlated with lower odds of cognitive decline. These results suggest that increased flavonoid intake maintains cognitive function in both men and women from the United States of America [69].

### 2.4. Studies on Mental Health 

Depression is one of the most common mental illnesses and complex mood disorders [70]. Moreover, people with dementia often suffer from depression [71,72]. A prospective cohort study including 82,643 women without a diagnosis of depression from the NHS (53–80 years old) and the NHSII (36–55 years old) showed an inverse correlation between the development of depression and citrus consumption. Moreover, maximum flavanone consumption (>64.2 mg/day) markedly reduced the risk of depression (by 10%). Thus, high flavonoid intake may reduce the risk of depression, especially among older women [56].

Although the neural circuitry underlying depression remains incompletely understood, depression could reduce BDNF levels in specific brain regions such as the hippocampus and prefrontal cortex [73]. Intriguingly, flavonoid consumption frequently increases BDNF levels in humans [74]. A single-blind, randomized controlled trial assessed the anti-depressant effects of citrus flavonoids [57]. Specifically, the authors evaluated the impact of consuming 380 mL flavonoid-rich orange juice (flavonoid content 600 ± 5.4 mg) daily for eight weeks on depressive symptoms in young individuals (aged 20–30 years). Unfortunately, the suppressive effect on depressive symptoms was not clear, and there was no clear significant difference between the high flavonoid group and the low flavonoid group after eight weeks. However, the authors observed a potential improvement in serum BDNF levels and both treatment regimens appeared to improve baseline scores of the Center for Epidemiological Studies Depression Scale (CES-D), a screening instrument for depression [57].

Untreated anxiety can seriously impair people’s daily lives. In recent years, the demand for methods to improve anxiety and mood disorders has risen [75]. In this context, a study investigated the effect of orange (*Citrus sinensis*) essential oils in dental clinics on anxiety and mood [58]. The authors divided 72 patients (aged 22–57 years) waiting for dental treatment into a control group (14 men and 23 women) and an odor group (18 men and 17 women). The orange odor was diffused in the odor group’s waiting room through an electric dispenser, while the control group’s room had no odor dispenser. The test assessed self-reported demographic and cognitive variables, trait and state anxiety, current pain, mood, alertness, and calmness. Women exposed to the orange scent had a lower level of anxiety, a more positive mood, and a higher level of calm than the controls. While citrus scent can relieve mental states such as anxiety [58], orange scent also effectively reduces anxiety associated with surgical removal of the mandibular third molar [76].

In addition, a randomized, parallel placebo-controlled trial compared the impacts of *Citrus* *aurantium* and lavender essential oils on the anxiety and agitation of conscious patients [59]. It included 150 subjects (aged 18–60 years) admitted to intensive care units randomly assigned to three groups: the lavender aromatherapy, *Citrus* *aurantium* aromatherapy, and placebo groups. The *Citrus* *aurantium* and lavender groups inhaled five drops of *Citrus* *aurantium* or lavender essential oils for 30 min, respectively. Meanwhile, the placebo group received five drops of saline for 30 min in addition to routine care. Immediately after and three hours after the intervention, the *Citrus aurantium* and lavender groups had significantly lower anxiety levels than the placebo group. No significant difference was observed between the lavender and *Citrus aurantium* groups. Although the *Citrus aurantium* and lavender improved restlessness/agitation more than the placebo did, no significant difference was observed between the three groups. These results suggest that lavender and *Citrus* *aurantium* aromatherapy can improve patient anxiety in intensive care units [59].

Cognitive and mental health impairment is a major feature of schizophrenia, which is generally refractory to treatment [77,78]. Recent studies have shown that early and effective interventions can lead to social and functional recovery in schizophrenia patients [79]. In an open-label pilot study, patients diagnosed with schizophrenia (*n* = 20) taking second-generation antipsychotics consumed a flavanone-rich bergamot polyphenol fraction (1000 mg/day) daily for eight weeks. This treatment significantly improved the Wisconsin Card Sorting Test “perseverative errors” and the Semantic Fluidity Test scores and marginally improved other cognitive outcomes [60].

## 3. Citrus Ingredients for Metabolic Function

Several observational studies have suggested that dyslipidemia, hypertriglyceridemia, and hyperglycemia are potential risk factors for the onset and/or progression of MCI and dementia [80,81]. Besides, patients with AD have significantly higher rates of hypertriglyceridemia and hyperglycemia, lower high-density lipoprotein (HDL), and higher low-density lipoprotein (LDL) concentrations [82]. In preclinical studies, several citrus flavonoids, such as nobiletin and hesperidin, have been reported to affect blood glucose and lipid metabolism via activating AMPK and PPARγ signaling pathways [83]. Epidemiological and clinical studies also suggest that citrus extracts positively affect metabolic functions (Table 2).

### 3.1. Effects of Citrus Components on Body Weight, Body Composition, and Lipid Profiles

Several clinical studies have shown the therapeutic effects of citrus and/or its extracts on body weight [84,85,86,87,88,89,90]. These studies indicated that citrus and/or its extracts had positive effects on weight loss. 

Dallas et al. (2008) investigated the lipolytic effect of Sinetrol^®^—a citrus-based polyphenolic dietary supplement—on human adipocytes, body fat, and biochemical mechanisms. Sinetrol^®^ contains 60% of polyphenols (e.g., catechin), 16.7% of flavanones (e.g., naringin), 2% of anthocyanins, and 3.6% of caffeine. Sinetrol^®^ promoted lipolytic activity [84]. The authors assessed its effect on fat mass and body weight through a randomized, double-blind, placebo-controlled trial. They assigned 20 subjects (aged 25–55 years) to two groups: the treatment group (*n* = 10) received four hard capsules containing 350 mg of Sinetrol^®^ and maltodextrin daily (1.4 g/day), while the placebo group (*n* = 10) received four hard capsules containing 350 mg of maltodextrin alone. In the treatment group, body fat decreased by 5.53% after four weeks and 15.6% after 12 weeks. Sinetrol^®^ may promote lipolysis and lower body mass index (BMI) by inhibiting cAMP-phosphodiesterase [84].

Furthermore, a randomized, double-blind, placebo-controlled trial investigated the efficacy and safety of Sinetrol^®^-XPur—a polyphenolic citrus dry extract—in weight management; metabolic parameters; and glycemic, inflammatory, and oxidative statuses [85]. Overweight subjects in the tested group (*n* = 47) received Sinetrol^®^-XPur twice daily with meals for 12 weeks and were compared with a placebo group (*n* = 48). Sinetrol^®^-XPur reduced waist and hip circumference and abdominal fat. It also reduced the levels of the inflammatory marker C-reactive protein (CRP). Moreover, it reduced malonaldehyde levels (indicating oxidative stress reduction) and increased superoxide dismutase and glutathione levels. In this intervention, no adverse effects were observed [85]. Similarly, two studies reported that ingesting 900 mg of Sinetrol^®^-XPur daily for 12 weeks improved body fat mass, body weight, and BMI more than a placebo did in obese and overweight individuals [86,87]. 

Aptekmann and Cesar (2010) investigated the effect of orange juice intake with aerobic training on serum lipids and physical characteristics of middle-aged (30–48 years) overweight women [88]. The intervention group (*n* = 13) consumed 500 mL of orange juice daily and performed one hour of aerobic exercise training three times a week for three months. The control group (*n* = 13) performed the same aerobic training program without consuming orange juice. The control lost an average of 15% of fat mass and 2.5% of body weight, while the intervention group lost 11% of fat mass and 1.2% of body weight. Besides, serum LDL levels decreased by 15% and HDL increased by 18% in the intervention group, while no significant changes were found in the control group [88].

A randomized, double-blind, placebo-controlled trial assessed the impact of CitruSlim, a blend of Citrus flavanone-*O*-glycosides and eurypeptides, on body composition and lipid parameters [89]. Researchers randomly separated 97 participants (aged 18–60 years) into a high CitruSlim dose (400 mg), low CitruSlim dose (200 mg), and placebo groups. The participants received three daily CitruSlim doses for 112 days. The high and low doses significantly reduced BMI (by 3.3% and 3.2%, respectively). Moreover, the intragroup analysis revealed that the low dose reduced fasting blood glucose after 112 days of intervention. However, it did not affect parameters associated with dyslipidemia and metabolic disturbances. These results suggest that CitruSlim effectively lowered body weight in obese subjects [89].

Another study documented the effect of dietary supplementation with CitriCholess—a bergamot extract-based formulation—on cholesterol levels, blood sugar, and body weight in older adults [90]. The 98 participants (mean age 65 years) with dyslipidemia were assigned to a CitriCholess (*n* = 48) or a placebo (*n* = 50) group. Each participant took two capsules with a meal, twice daily for 12 weeks. CitriCholess supplements lowered triglycerides (TG), total cholesterol (TC), and LDL levels and improved body weight, BMI, and waist circumference. In addition, a gender analysis revealed that the HDL reduction was stronger in women than in men. These results suggest that 12-week CitriCholess supplementation enhances lipid metabolism and helps weight management in older adults with dyslipidemia [90].

Furthermore, a randomized, double-blind crossover study assessed the impacts of consuming orange juice containing either normal (NPJ, 299 mg/day) or high (HPJ, 741.5 mg/day) polyphenols concentrations on the antioxidant, oxidative stress biomarkers, and metabolic syndrome clinical markers levels in 100 non-smoking obese subjects [91]. NPJ and HPJ both decreased urinary 8-hydroxy-2′-deoxyguanosine, 8-iso-prostaglandin F2α, erythrocyte catalase, and glutathione reductase activities. They also decreased BMI, belly circumference, and leptin levels. Moreover, chronic NPJ intake decreased systolic and diastolic blood pressures. Finally, NPJ and HPJ both protected against lipid peroxidation and DNA damage, modified several antioxidant enzymes, and reduced body weight in obese adults [91].

Animal and in vitro experiments reported that Japanese sudachi peel (containing sudachitin) might positively affect inflammation, hyperlipidemia, and obesity [96,97]. Shikishima et al. (2021) investigated the effect of sudachitin on visceral fat content in a randomized, double-blind, placebo-controlled trial [92]. They randomly assigned 41 subjects (aged 30–65 years) with BMI of 23–30 kg/m^2^ to a sudachi peel extract powder intake group (4.9 mg of sudachitin taken daily for 12 weeks, *n* = 21) or a placebo group (*n* = 20). Sudachi peel extract powder significantly improved the ratio of visceral fat to subcutaneous fat compared with the placebo and reduced belly circumference, a metabolic syndrome marker. Meanwhile, glycemia and lipid profiles remained unaltered [92].

Although various clinical trials have reported the lipid metabolism and weight-regulating function of citrus extract ingestion, it remains controversial [98,99,100]. Thus, revealing the effect of citrus compounds intake on body weight and lipid metabolism may require further randomized controlled trials.

### 3.2. Liver Steatosis and Non-Alcoholic Fatty Liver Disease

Liver steatosis and fibrosis are emerging risk factors for multiple extrahepatic health conditions, including dementia [101]. Researchers assessed the effect of bergamot extract on fatty liver disease [102] through a placebo-controlled, double-blind clinical trial including 102 subjects (aged 30–75 years) with liver steatosis. The intervention group received 300 mg/day of a dietary supplement containing a bergamot polyphenol fraction and *Cynara cardunculus* extract for 12 weeks, while the control group received a daily placebo. Liver fat content, serum transaminases, lipids, and glucose were evaluated at the baseline and at week 12. Participants taking dietary supplements had a significantly lower liver fat content than placebo group members. The rate of decrease in the controlled attenuation parameter (CAP) score was significant in obese patients, overweight/obesity patients, and women. After adjusting for weight changes, the rate of decrease in CAP score was significant only in people over 50 years. Thus, bergamot and wild cardoon extract could become the cornerstone of liver steatosis treatments [102].

Non-alcoholic fatty liver disease (NAFLD) is a major aging-related disorder and a risk factor for diabetes, obesity, and cardiovascular diseases [103,104]. NAFLD is exacerbated by type 2 diabetes mellitus (T2DM) co-occurrence, which enhances the inflammatory and fibrotic processes. Furthermore, NAFLD may affect cognitive decline in older people [105,106], although this association is controversial [107]. A research group studied the impact of Bergacyn, a preparation derived from the combination of bergamot polyphenol fraction (BPF) and *Cynara cardunculus* (CyC). Eighty patients with a history of T2DM and NAFLD for at least 12 months received BPF (300 mg/day) and CyC (300 mg/day) separately or in combination with a finely milled and co-milled excipient containing 300 mg bergamot albedo fiber (Bergacyn; 300 mg/day) or a placebo. Serum measurements and liver ultrasound analyses showed that BPF and CyC consumption significantly improved NAFLD biomarkers in patients with T2DM. Furthermore, this effect was correlated with significant improvements in inflammatory and oxidative stress biomarkers, such as superoxide dismutase, glutathione peroxidase, tumor necrosis factor (TNF)-α, and malondialdehyde levels [108]. 

### 3.3. Glycemia

The citrus fruit of Tanaka (*C. junos*), also recognized as Yuzu, is a citrus fruit readily available in East Asia, rich in phenol and vitamin C [109,110]. It contains the phenolic compounds hesperidin and naringin [109], known for improving glycemia [111]. Hwang et al. (2015) investigated the effect of *C. junos* peel extract on glycemic responses in subjects with impaired fasting glucose [93]. After eight weeks of intervention (4250 mg/day), the active group indicated significantly lower fasting plasma glucose, fasting plasma insulin levels, and homeostatic model assessment-insulin resistance than the placebo group. The intervention group also had slightly reduced C-peptide levels but no significant improvement in postprandial glucose levels compared with the placebo group. These results suggest that *C. junos* peel extract improves fasting glycemic indices and has an anti-diabetic effect in individuals with impaired fasting glucose [93]. According to animal experiments, the hypoglycemia may be due to an increase in glucose uptake through increased insulin action in peripheral tissues [112].

### 3.4. Studies on Bone Metabolism

In humans, bone mineral density peaks at approximately 25–30 years of age and gradually decreases thereafter [113,114]. This bone mineral density loss and increased fracture risk, named osteoporosis, is common in older people [115,116]. Healthy individuals have balanced bone formation and resorption. However, aging often increases bone resorption [117]. Age-related bone loss is found in both men and women, but perimenopausal women often experience a rapid bone loss phase [118,119]. Patients with AD have high bone loss rate and fracture incidence, dramatically impacting their quality of life [120,121]. 

Preclinical studies indicated that hesperidin prevents bone loss and bone metabolic markers in ovariectomized animals [122,123]. Martin et al. (2016) evaluated the impact of hesperidin intake with or without calcium supplementation on bone calcium retention in postmenopausal women [94]. In this randomized, double-blind crossover trial, 12 healthy postmenopausal women received hesperidin (500 mg) with or without calcium supplementation. Bone calcium retention was evaluated by urinary excretion of a rare isotope from bones. Hesperidin and the calcium supplement improved net calcium bone retention by 5.5% (*p* < 0.04). Thus, calcium supplementation, combined with hesperidin, effectively preserved bone health in postmenopausal women [94].

To assess the synergistic effect of the kudzu flower and mandarin peel extract mixture (KM) on bone metabolism marker levels in menopausal women, Kim et al. (2020) randomly assigned participants to a KM (1150 mg/day) group and a placebo group (*n* = 84) [95]. KM intake for 12 weeks ameliorated bone turnover marker levels, notably reducing the levels of the bone resorption marker C-telopeptide fragment and marginally increasing the bone formation marker osteocalcin compared with the placebo. No serious side effects and hormonal changes were observed in the KM or placebo groups.

## 4. Stroke and Vascular Function

Blood pressure is an important predictor of cardiovascular health, as lower blood pressure is associated to better vascular health [124]. Reducing systolic blood pressure can reduce stroke mortality [124]. Dementia is a frequent outcome after stroke and increases mortality and disability risk [125,126]. In general, older people, who are at the highest risk of stroke, are also at increased risk of dementia in the absence of a stroke [127]. The protective effect of citrus flavonoids in cerebrovascular disease and cardiovascular health is well-established and seems to extend to stroke incidence [128,129,130]. For example, citrus flavonoids hesperidin and nobiletin can activate PPARγ signaling and reduce diastolic pressure and mean arterial pressure in diabetic rats [128,129,130]. Therefore, it is worth mentioning that citrus flavonoid intake affects blood pressure and vascular function, reducing the risk of stroke, making them particularly relevant to neurodegenerative diseases (Table 3).

A prospective cohort study followed 69,622 women (aged 30–55 years) from the NHS for 14 years to assess the association between dietary flavonoid intake and stroke risk [131]. Flavonoid consumption was not inversely correlated with stroke risk, but the ischemic stroke risk was 19% lower in women with high flavanone intake (>62.95 mg/day) than in women with low intake (<13.72 mg/day). Furthermore, a marginal inverse correlation was observed between citrus juice consumption and ischemic stroke risk. However, these apparently protective effects did not affect hemorrhagic stroke risks [141,142].

Goetz et al. (2016) utilized the Reasons for Geographic and Racial Differences in Stroke (REGARDS) database to identify a potential correlation between flavonoid intake and accidental ischemic stroke in a heterogeneous cohort [132]. The authors determined the flavonoid intake of 20,024 participants aged 45 years or more from the FFQ and followed it for 6.5 years. After multivariate adjustment, higher flavanone intake (>48 mg/day) was inversely associated with ischemic stroke incidence compared with lower intake (<3.9 mg/day), and citrus juice intake similarly reduced risk (hazard ratio: 0.69) [132].

Oxidized LDL (Ox-LDL) plays a central role in the initiation and progression of atherosclerotic plaques and causes progressive diseases, such as ischemic stroke and other atherosclerotic cardiovascular diseases [143,144]. Therefore, lowering Ox-LDL blood levels can reduce cardiovascular event incidence in high-risk individuals [145,146]. In a randomized, double-blind, controlled study, 23 untreated participants (mean age of 41.9 years) with cardiovascular risk (TC > 200 mg/dL and LDL > 130 mg/dL) consumed flavonoid-rich hydroethanolic extract (Citrolive™) daily (1000 mg) for 90 days. The authors observed significantly reduced Ox-LDL levels and LDL-oxidase/LDL ratio and increased serum paraoxonase activity relative to controls [133]. In another clinical trial involving 96 healthy individuals (51 intervention, 45 placebo), an eight-week supplementation with Citrolive™ (1000 mg/day) ameliorated blood pressure, endothelial function (assessed by flow-mediated vasodilation), lipid metabolism-associated parameters (TC, LDL, LDL-oxidase, oxidized/reduced glutathione ratio, protein carbonyls, IL-6), and improved antioxidant and anti-inflammatory properties [134].

A randomized, placebo-controlled, double-blind study evaluated the effect of NP06-1, a combination of two botanical extracts of *Phellodendron amurense* bark and *Citrus sinensis* peel, on cardiovascular risk factors, lipid levels, and fasting blood glucose levels [135]. Eight weeks of NP06-1 treatment improved lipid levels, markedly decreased TG and LDL levels, and notably increased HDL levels. In addition, NP06-1 positively affected weight and symptoms of osteoarthritis of the knee and had anti-inflammatory effects (measured by quantifying CRP) [136].

A randomized, double-blind controlled clinical trial evaluated the impacts of hesperidin supplementation on blood pressure and inflammatory markers in T2DM patients [137]. In this trial, 64 patients (aged 30–65 years) received 500 mg of hesperidin or placebo capsules daily for six weeks. In the hesperidin group, systolic blood pressure, mean arterial blood pressure, and high-sensitivity CRP (hs-CRP) levels decreased, whereas serum total antioxidant capacity increased compared with the baseline. In addition, the placebo and hesperidin groups had significantly different mean percent changes of systolic blood pressure, diastolic blood pressure, mean arterial blood pressure, serum total antioxidant capacity, and inflammatory markers (e.g., TNF-α, IL-6, and hs-CRP) after intervention in adjusted models. These results suggest that chronic hesperidin intake exerts antihypertensive and anti-inflammatory effects in T2DM patients [137]. Additionally, other clinical trials have shown that taking orange juice, which is rich in flavonoids, significantly lowers blood pressure [138] and pulse pressure [139] while improving flow-mediated dilation [140].

## 5. Other Functions and Future Perspectives

### 5.1. Circadian Rhythms 

Circadian rhythms are bioactive rhythms driven by the circadian clock in several organs and are the basic regulatory mechanism for various physiological functions [147]. Disturbances in circadian rhythms are associated with the development of many illnesses, including dyslipidemia, obesity, inflammation, and cognitive decline [148,149]. In addition, circadian disruption is common in older adults and more severe in patients with neurodegenerative diseases such as AD [148,149]. In the last decade, several preclinical studies have documented how citrus PMFs (e.g., nobiletin) regulate the biological clock and circadian rhythms [150,151,152,153,154]. Interestingly, nobiletin regulates circadian rhythms and improves metabolic disease, neuroinflammation, and cognitive function in animal models [151,153,154,155]. The improvement of cognitive function by citrus flavonoids may involve the regulation of circadian rhythms. However, to our knowledge, no clinical studies have examined the relationship between citrus consumption and biological clock regulation. The effects of citrus polyphenols on the circadian clock may need to be clinically evaluated.

### 5.2. Gut Microbiota

The gut microbiota has emerged as a crucial factor in many diseases, including neurodegenerative diseases, and offers potential new therapeutic options [156,157,158]. Moreover, the gut microbiota metabolizes citrus flavonoids—such as hesperidin, naringin, and nobiletin—into phenolic and aromatic splitting heterocompounds, enhancing their bioavailability [159]. The increased bioavailability enhanced the efficacy of citrus flavonoids in animal models [160,161]. In preclinical studies, long-term ingestion of nobiletin has been reported to have an anti-obesity effect by altering the activity of the intestinal microbiota [160]. Nobiletin has also been shown to promote thermogenesis of brown and beige adipose tissue and reduce body weight in mice fed a high-fat diet by affecting the formation of the gut microflora [161]. Interestingly, one interventional study showed that consuming 300 mL of orange juice for 60 days modulated the gut microbiota and simultaneously improved blood glucose and lipid profiles [162]. Given that the gut microbiota is implicated in various diseases, including dementia and obesity, further clinical studies may be warranted.

## 6. Conclusions

This review explored the effects of citrus components on brain health and related functions. Numerous clinical and epidemiological studies have demonstrated the benefits of acute and chronic consumption of citrus components on cognitive functions for healthy or preclinical individuals and patients. Another great advantage of citrus flavonoids is their safety. Indeed, high-dose and chronic intake of citrus peels and extracts have no serious adverse effects on humans or animals [48,49,50,163,164,165]. Therefore, citrus ingredients are safe to use in diets or supplements for potential neurological, cardiac, and/or metabolic benefits. In addition, citrus ingredients are potential adjuncts to therapeutic agents against various diseases such as AD. However, the heterogeneity of the reported evidence and the variety of citrus types and amounts explored mean that strengthening the clinical findings on the effects of citrus extracts on dementia and related disorders requires further research.

## Figures and Tables

**Table 1:** Effect of citrus peels, extracts, and juices on cognitive function and mental health.

Intervention or Cohort Analysis	Dosage or Frequency	Study Design	Subjects	Duration	Reference
Cognitive health					
Nobiletin-containing test food (Nobilex®)	3 capsules (containing 30 mg nobiletin and 17.4 mg tangeretin)	Randomized, double-blind, placebo-controlled trial	Healthy elderly individuals (*n* = 108), aged over 65	16 weeks	[48]
Nobiletin-rich ponkan peel powder and *perilla* seed oil	1.12 g ponkan peel powder (containing 2.91 mg nobiletin) and 1.47 mL *perilla* seed oil	Randomized, double-blind, parallel-armed trial	Healthy elderly individuals (*n* = 49), aged 60–85	12 months	[49]
Nobiletin-rich citrus peel extract	30 g of citrus peels boiled in 500 mL of water and concentrated to 300 mL	Randomized, double-blind, placebo-controlled trial	Alzheimer's disease patients taking donepezil (*n* = 11)	12 months	[50]
High flavanone orange juice	500 mL (flavanone 305 mg)	Randomized, double-blind, placebo-controlled crossover trial	Healthy elderly people (*n* = 37), aged 60–81	8 weeks	[51]
Flavonoid-rich orange juice	240 mL (flavonoid 272 mg)	Randomized, double-blind, placebo-controlled, crossover trial	Healthy middle-aged adults (*n* = 24), aged 30–65	Acute	[52]
Flavanone-rich orange juice	500 mL (flavanone 70.5 mg)	Randomized, single-blind, cross over trial	Healthy young adults (*n* = 40), aged 18–30	Acute	[53]
Auraptene-rich Kawachi Bankan extract	125 mL (auraptene 6 mg)	Randomized double-blind, placebo-controlled trial	Healthy elderly people (*n* = 82), aged 62–80	24 weeks	[54]
Daily citrus intake	3–4 or more times per week	Retrospective cohort study	13,373 adults (over 65 years)	5.7 years follow up	[55]
Mental health					
Daily citrus intake	Total dietary flavonoid intake	Prospective cohort study	82,643 women, aged 36–55 and 53–80	10 years follow up	[56]
Flavonoid rich orange juice	380 mL (flavonoid 600 mg)	Randomized single-blind trial	Depressive symptoms in young individuals (*n* = 40), aged 20–30	8 weeks	[57]
*Citrus sinensis* essential oils	Diffused through electric dispenser	Randomized trial	Patients undergoing treatment at a dental (*n* = 72), aged 22–57	Acute	[58]
*Citrus aurantium* or lavender essential oils	Inhalation of 5 drops of lavender or *Citrus aurantium* essential oils for 30 min	Randomized, parallel group placebo-controlled trial	Subjects admitted to intensive care units (*n* = 150), aged 18–60	Acute	[59]
Flavanone-rich bergamot polyphenol fraction	1000 mg	Open-label pilot study	Patients diagnosed with schizophrenia (*n* = 20), aged 20–58	8 weeks	[60]

**Table 2:** Beneficial effect of citrus peels, extracts, and juices on body weight, lipid profiles, fat content, and bone health.

Intervention or Cohort Analysis	Dosage or Frequency	Study Design	Subjects	Duration	Reference
Body weight, lipid profiles, and fat content					
Citrus-based polyphenolic dietary supplement, SINETROL^®^	4 capsules (1400 mg)	Randomized, double blind, placebo-controlled trial	Overweight subjects (*n* = 20), aged 25–55 (BMI 27–33)	12 weeks	[84]
Citrus-based polyphenol extract, Sinetrol^®^-Xpur	2 tablets (900 mg)	Randomized, double-blinded, controlled study	Overweight subjects (*n* = 95) or overweight/obese participants (*n* = 100)	12 weeks	[85,86,87]
Orange juice with aerobic training	500 mL of orange juice and 1 h aerobic training 3 times a week	Randomized, controlled study	Overweight (weighing 75.5 ± 14.2 kg) women (*n* = 26), aged 30–48	3 months	[88]
Citrus flavanone-O-glycosides and eurypeptides, CitruSlim	200 mg or 400 mg	Randomized, double-blind, placebo-controlled trial	97 participants (ages 18–60)	112 days	[89]
Bergamot extract-based formulation, CitriCholess	2 capsules (containing 500 mg Citrus bergamia Risso extract and others)	Randomized, double-blind, placebo-controlled trial	98 participants (mean age 65)	12 weeks	[90]
Normal or high polyphenol concentration in orange juice	500 mL (containing 299 or 741.5 mg polyphenols)	Randomized, double-blind crossover study	Non-smoking obese subjects (*n* = 100), aged 18–65	12 weeks	[91]
Sudachi peel extract powder	1050 mg purified sudachi extract (including 4.9 mg sudachitin)	Randomized, double-blind, placebo-controlled trial	Mild overweight (BMI 23–30 kg/m^2^) subjects (*n* = 41), aged 30–65	12 weeks	[92]
*Citrus junos Tanaka* peel extract	4250 mg	Randomized, double-blind, crossover, placebo-controlled clinical trial	Subjects with impaired fasting glucose (*n* = 40), average 52.75 years	8 weeks	[93]
Bone health					
Hesperidin and calcium supplement	500 mg hesperidin with or without calcium supplement	Randomized, double-blind crossover design	Healthy postmenopausal women (*n* = 12), mean age 66.3 years	350 days	[94]
Extract mixture of kudzu flower and mandarin (*Citrus unshiu Markovich*) peel	1150 mg	Randomized controlled parallel-armed design	Peri- or post-menopausal women (*n* = 84), aged 45–60	12 weeks	[95]

**Table 3:** Effect of citrus peels, extracts, and juices on stroke risks and vascular functions.

Intervention or Cohort Analysis	Dosage or Frequency	Study Design	Subjects	Duration	Reference
Daily flavanone intake	>62.95 mg/day	Prospective cohort study	69,622 women, aged 30–55	14 years follow up	[131]
Daily flavonoid intake	>48 mg/day	Prospective cohort study	20,024 subjects, aged 45 years or older	6.5 years follow up	[132]
Flavonoid-rich hydroethanolic extract Citrolive™	2 capsules (1000 mg)	Randomized, double-blind, controlled study	23 participants (mean age 41.9) with cardiovascular risk (cholesterol level > 200 mg/dL and LDL > 130 mg/dL)	3 months	[133]
Flavonoid-rich hydroethanolic extract Citrolive™	2 capsules (1000 mg)	Randomized, double-blind, placebo-controlled study	Healthy individuals (*n* = 96), aged 40–75	8 weeks	[134]
Extracts of *Phellodendron amurense* bark and *Citrus sinensis* peel, NP06-1	4 capsules (1480 mg)	Randomized, double-blind, placebo-controlled pilot study	Normal weight (BMI 18.9–24.9) or overweight (BMI 25–40) subjects (*n* = 80), aged 25–60	8 weeks	[135,136]
Hesperidin supplementation	500 mg	Randomized double-blind controlled clinical trial	Patients with type 2 diabetes mellitus (*n* = 64), aged 30–65	6 weeks	[137]
Orange juice or hesperidin	500 mL orange juice (292 mg hesperidin and 47.5 mg narirutin) or pure hesperidin 292 mg	Randomized, controlled, crossover study	Healthy overweight men (*n* = 24), aged 50–65	4 weeks	[138]
Orange juice or hesperidin-enriched orange juice	500 mL (containing 345 mg or 600 mg hesperidin)	Randomized, parallel, double-blind, placebo-controlled trial	Pre- or stage-1 hypertensive individuals (*n* = 159), aged 18–65	12 weeks	[139]
Blood orange juice	400 mL (hesperidin and narirutin concentration were 80.2 and 9.5 mg/dL)	Randomized, controlled, single-blind, crossover trial	Overweight or obese subjects (*n* = 15) (BMI: 28.3 ± 3.1 kg/m^2^), aged 20–45	2 weeks	[140]

## Data Availability

Not applicable.
